# Perioperative Nursing Interventions for a Morbidly Obese Patient Undergoing a Cesarean Section in the Ramp Position: A Case Report

**DOI:** 10.7759/cureus.85673

**Published:** 2025-06-09

**Authors:** Kayoko Kusuhara, Reika Tachibana, Ryosuke Kumashiro, Kentaro Hara

**Affiliations:** 1 Department of Nursing, National Hospital Organization (NHO) Nagasaki Medical Center, Nagasaki, JPN; 2 Department of Fundamental Nursing, Kumamoto University, Kumamoto, JPN; 3 Clinical Research Center, National Hospital Organization (NHO) Nagasaki Medical Center, Nagasaki, JPN

**Keywords:** cesarean section (cs), ectopic pancreas, heterotopic pancreas, morbid obesity, perioperative nursing, ramp position, simulation-based preparation, sleeve gastrectomy, submucosal lesions

## Abstract

This case report describes a multidisciplinary perioperative nursing intervention for a morbidly obese woman in her 30s (BMI 50.9) undergoing elective cesarean section. The patient had severe obstructive sleep apnea and orthopnea, making the conventional supine position unsafe. A preoperative simulation involving obstetricians, anesthesiologists, surgical nurses, and clinical engineers was conducted to evaluate the feasibility of performing the surgery in the ramp position. The team also prepared emergency response protocols using a photo-based manual and preassembled equipment. During surgery, the patient was successfully positioned in the ramp position and underwent spinal anesthesia. Due to inadequate pain control, sedation was initiated, which led to respiratory depression requiring bilevel positive airway pressure (BiPAP) support. No intraoperative or postoperative complications, such as pressure injuries or nerve damage, were observed. The patient reported no psychological distress related to the positioning or simulation. This case highlights the importance of simulation-based planning and interprofessional collaboration in ensuring surgical safety for high-risk patients. Surgical nurses played a central role in coordinating the simulation and intraoperative care, emphasizing their contribution to perioperative team performance. This report offers a practical model for managing complex obstetric cases involving morbid obesity through nursing-led simulation and tailored patient-centered care.

## Introduction

Obesity is a growing global health concern, significantly impacting the number of obese patients undergoing surgical procedures [1,2]. Obesity has been identified as a risk factor for postoperative complications, including the development of pressure injuries [3,4]. Therefore, in surgical care for obese patients, perioperative nursing interventions are essential to reduce these risks and ensure patient safety throughout the surgical period.

A cesarean section is typically performed in the supine position. However, this position can lead to hypotension due to decreased venous return caused by inferior vena cava compression, particularly in pregnant patients [5]. In the present case, the patient was morbidly obese (BMI 50.9), and due to concerns regarding anesthesia induction and compromised respiratory function, a conventional supine position was deemed inappropriate.

Obesity is a well-established risk factor for perioperative complications. Previous studies have reported that cesarean delivery in patients with a BMI of >40 is associated with significantly increased rates of wound infection, respiratory compromise, and anesthetic complications [6,7]. The ramp position, which involves elevating the upper body by approximately 20 to 30 degrees, has been reported to be effective in improving airway patency and respiratory management in morbidly obese patients [6,7]. Nevertheless, this positioning can also interfere with the surgical field, especially in lower abdominal surgeries such as a cesarean section [8,9].

This case report describes a multidisciplinary perioperative nursing intervention for a morbidly obese pregnant patient who underwent a cesarean section in the ramp position. A preoperative simulation, collaborative planning, and respiratory and positioning strategies were employed to optimize safety. In this case, the selected angle was based on prior findings, aligning with evidence-based practices for optimizing respiratory function during anesthesia in obese parturients. This report highlights the importance of interprofessional collaboration and perioperative nursing care in enhancing surgical outcomes for high-risk patients.

## Case presentation

The patient was a 30-year-old woman, gravida 4 para 3, with a height of 154 cm, a weight of 121 kg, and a body mass index (BMI) of 50.9, classifying her as morbidly obese. She had a history of three previous cesarean sections and was scheduled for an elective cesarean delivery at 38 weeks of gestation due to her repeat cesarean sections and obesity-related risks. Her medical history also included severe obstructive sleep apnea syndrome (SAS), cardiac enlargement, and orthopnea; however, she was not hypertensive during pregnancy or at the time of surgery. She was dependent on continuous positive airway pressure (CPAP) therapy during sleep and expressed strong anxiety about airway management and the use of general anesthesia. To address this, perioperative nurses and anesthesiologists engaged in repeated, empathetic communication with the patient, explaining the surgical and anesthetic procedures in detail. This approach helped alleviate her anxiety and foster trust in the care team.

During preoperative assessment, the anesthesiology team determined that conventional supine positioning would pose a risk of respiratory distress and airway obstruction. Consequently, a plan was made to perform the cesarean section in a ramp position, with the upper body elevated to support respiratory function. However, this approach risked limiting access to the lower abdominal surgical field, prompting the team to organize a multidisciplinary simulation one week before surgery. The simulation included the patient, obstetricians, anesthesiologists, surgical nurses, and a clinical engineer.

**Figure 1:**
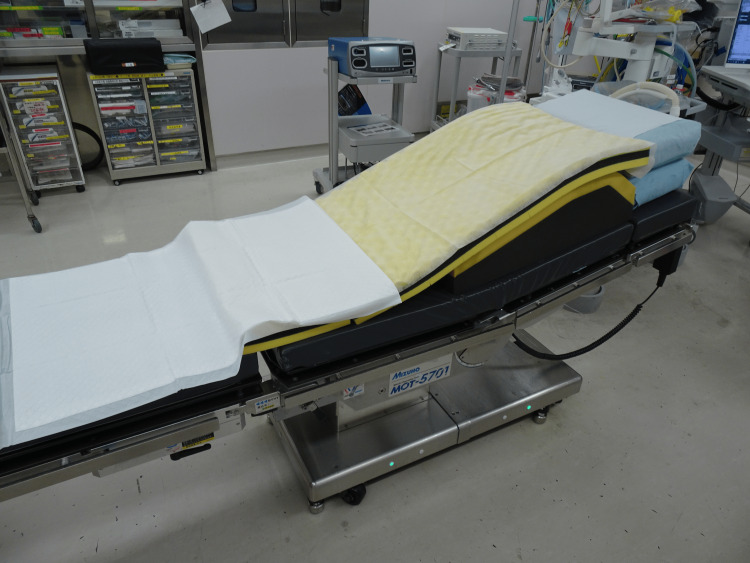
Operating table setup for the ramp position Layered positioning pads, including PureFix® (HOPES Co., Ltd., Tokyo, Japan) and SliceFix® (Paramount Bed Co., Ltd., Tokyo, Japan), were used on the Mizuho MOT-5701 operating table to achieve an approximate 20° elevation of the upper body. This configuration was adjusted based on patient feedback to alleviate dyspnea during the simulation.

The simulation was conducted using a Mizuho MOT-5701 operating table (Mizuho Corporation, Tokyo, Japan). PureFix® (HOPES Co., Ltd., Tokyo, Japan) and SliceFix® (Paramount Bed Co., Ltd., Tokyo, Japan) pads were layered on the table to achieve head elevation. Initially, the table was set flat, but the patient experienced dyspnea. An additional 10° head-up tilt was applied, resulting in a 20° inclination, which relieved her symptoms (Figure 1).

**Figure 2:**
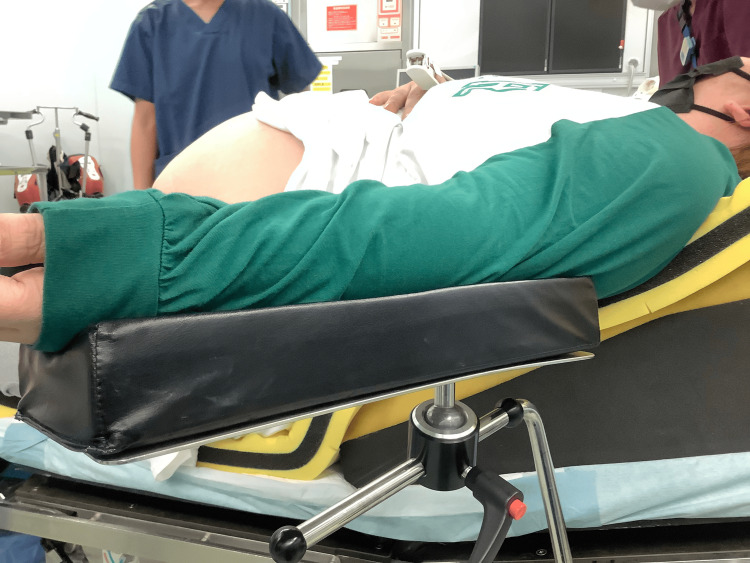
Upper limb fixation using adjustable arm supports Quick Fix Arm Support® (Baxter Ltd., Tokyo, Japan) devices were utilized to maintain the upper limbs in a comfortable abducted position. This avoided excessive shoulder extension and prevented height discrepancies caused by conventional arm boards.

For upper limb positioning, Quick Fix Arm Support® (Baxter Ltd., Tokyo, Japan) devices were used to ensure comfortable shoulder abduction without hyperextension (Figure 2).

**Figure 3:**
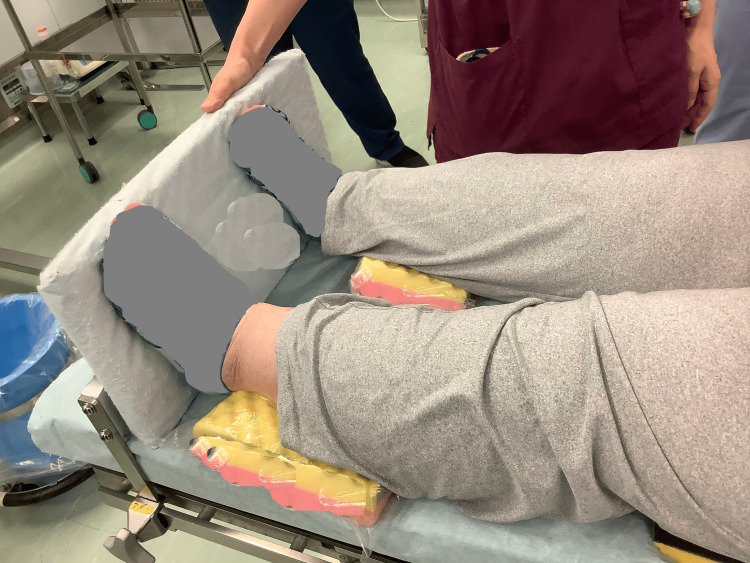
Final lower body positioning and support configuration Softnurse® pads (ALCARE Co., Ltd., Tokyo, Japan) were placed beneath the thighs and calves, and footboards were used to prevent sliding. This setup ensured patient stability, pressure offloading, and surgical field accessibility while accommodating respiratory support.

Softnurse® pads (ALCARE Co., Ltd., Tokyo, Japan) were placed beneath the thighs and calves, and footboards were installed to prevent sliding. Once positioning was complete, the obstetrician confirmed that the surgical field remained accessible despite the inclination (Figure 3).

The simulation also included an assessment of positioning for neuraxial anesthesia. The patient was seated on the table, and anesthesiologists used ultrasound to identify intervertebral spaces. To prepare for potential respiratory complications, the patient's CPAP device was brought into the operating room, and a bilevel positive airway pressure (BiPAP) unit was set up by the clinical engineer. In preparation for potential airway compromise, the anesthesiologists developed an emergency airway management plan, which included ready access to advanced airway equipment such as video laryngoscopes, supraglottic airway devices, and a tracheostomy set. These plans were shared in advance with the surgical and nursing teams to ensure coordinated response readiness in the event of respiratory failure.

Because the surgery was scheduled for early the following week, the team also prepared for potential emergency delivery over the weekend. A photo-based manual documenting positioning techniques, required equipment, and anesthesia plans was shared with staff, and all supplies were preassembled in a designated area.

On the day of surgery, the operating table and equipment were prepared exactly as practiced. Spinal anesthesia combined with epidural anesthesia (hyperbaric bupivacaine) was administered under ultrasound guidance in the seated position. The patient was then placed in the ramp position with a slight Trendelenburg tilt, resulting in an upper body elevation of approximately 14°. This angle was selected based on prior simulation trials and existing literature suggesting that a 10-30° elevation improves airway patency in morbidly obese patients [6,7], while also maintaining adequate surgical access.

Once sensory loss below T4 was confirmed, surgery proceeded. Throughout the procedure, CPAP was used continuously. The patient remained hemodynamically stable, with SpO₂ levels maintained between 94-96%, a respiratory rate of 14-18 breaths per minute, and end-tidal carbon dioxide (EtCO₂) levels between 35 and 42 mmHg. However, about one hour into the surgery, she began experiencing pain, which was managed with additional epidural and intravenous medications. After three hours, sedation with propofol and dexmedetomidine became necessary due to persistent pain. As sedation deepened, BiPAP support was initiated to address signs of respiratory depression. The final level of sensory block was estimated at T6.

Manual upward traction of the abdominal wall by the assistant obstetrician was required for optimal exposure, but no additional retractors or instruments beyond standard cesarean equipment were needed. Five obstetricians participated in the procedure. Two of them were responsible for manual upward traction of the abdominal wall to optimize the surgical field. There were no staff changes during the procedure, ensuring consistency in the surgical team throughout the operation. Delivery occurred approximately 30 minutes after the skin incision, and the total surgical time was 2 hours and 33 minutes. This extended duration reflects the technical challenges associated with wound closure in morbidly obese patients, which typically prolongs the procedure compared to the average cesarean section time of approximately 60-90 minutes. The prolonged surgical duration was primarily due to difficulty in abdominal wall closure, which is frequently encountered in morbidly obese patients.

The anesthesia administration began at 11:43 a.m., followed by surgical preparation and positioning. The skin incision was made at 12:35 p.m., and delivery occurred at 1:03 p.m. The operation concluded at 3:08 p.m., with the patient fully recovered and transferred to the ward at 3:45 p.m.

The infusion of propofol and dexmedetomidine was discontinued at the end of the surgical procedure and was not continued postoperatively.

Postoperatively, the patient had no pressure injuries or neurological complications. She reported no increased anxiety or burden from the simulation process and expressed appreciation for the individualized care and team preparation.

For patient transfer and mobilization, a bariatric-compatible stretcher and bed were used. The transfer was carried out by a team of six staff members, including surgical nurses and ward personnel, with continuous monitoring to prevent injury and ensure safety.

Postoperative recovery was uneventful. The patient was able to initiate oral intake the day after surgery and began ambulating on postoperative day two. She was monitored for respiratory stability and wound healing and demonstrated stable vital signs throughout. She was discharged on postoperative day six after confirming readiness for home care and independent activity.

## Discussion

This case report describes a perioperative nursing intervention in a morbidly obese pregnant patient (BMI 50.9) undergoing cesarean section in the ramp position. The patient’s comorbidities, including severe obstructive SAS and orthopnea, presented unique challenges to positioning, anesthesia management, and respiratory support. The abbreviation SAS is defined here and used consistently throughout the manuscript. Conventional supine positioning was considered unsafe due to the risk of respiratory compromise and airway obstruction, prompting the need for an individualized, multidisciplinary care approach [5].

The ramp position has been reported as effective in improving respiratory mechanics and airway management in obese patients during anesthesia induction [6,7]. Previous case reports and observational studies have focused primarily on anesthetic considerations and airway management in the ramp position [6-9]; however, detailed accounts of intraoperative nursing strategies and simulation-based perioperative planning in cesarean sections remain limited.

In contrast to prior reports that briefly mention positioning or airway adjustments, this case highlights the role of surgical nurses in leading a structured preoperative simulation, incorporating practical adjustments such as elevation angle optimization, padding, emergency device setup, and interdisciplinary rehearsal. This level of perioperative planning, particularly involving nursing leadership, offers a reproducible model for managing complex obstetric patients with severe obesity.

To balance the need for respiratory support and surgical accessibility, a preoperative simulation was conducted involving obstetricians, anesthesiologists, surgical nurses, and clinical engineers. This proactive simulation allowed for the refinement of patient positioning, equipment preparation, and role assignment, ensuring both respiratory safety and surgical access.

Notably, the patient required sedation due to inadequate pain control despite combined spinal-epidural anesthesia. This led to respiratory depression, necessitating the use of BiPAP. Anticipating such a scenario, the team had pre-arranged the availability of CPAP and BiPAP equipment in the operating room, demonstrating the value of comprehensive preparation. Importantly, the preoperative simulation facilitated the development of shared mental models among team members, enabling them to anticipate each other’s actions and respond cohesively. Shared mental models are known to support team performance and reduce error in high-acuity surgical environments [10,11], and have been recognized as essential coordinating mechanisms in interprofessional clinical settings [12].

Additionally, the ramp position itself plays a critical role in airway management for morbidly obese patients. It improves upper airway patency and facilitates both spontaneous breathing and potential mask ventilation or intubation. Although general anesthesia was avoided in this case, the anesthesia team developed contingency plans for possible airway compromise, including readiness for awake intubation or rapid sequence induction. These steps reflect the necessity of integrated airway strategies when managing high-risk patients in non-standard surgical positions.

From a nursing perspective, the surgical nurses played a pivotal role in coordinating preoperative simulation, constructing the customized operating table setup, and preparing for potential emergency procedures. The preparation of a photo-based manual and centralization of required equipment ensured that the operating room staff could respond promptly even if the patient had required surgery over the weekend. Importantly, there were no postoperative complications such as pressure injuries or nerve damage, and the patient reported no distress associated with the simulation or surgical positioning. These outcomes reflect the effectiveness of nursing-led planning and the value of interprofessional collaboration.

This case underscores the importance of individualized perioperative care for high-risk patients and highlights how surgical nurses can lead simulation-based planning to enhance patient safety and operative success. While performing a cesarean section in the ramp position is rare, particularly in morbidly obese patients, this case demonstrates that with careful preparation and multidisciplinary engagement, safe and effective outcomes can be achieved. Only a few case reports or small studies have explored the use of ramp positioning during cesarean delivery, and most available literature focuses on its utility during anesthesia induction [7]. Our case contributes novel insights by demonstrating its application throughout the perioperative period.

**Figure 4:**
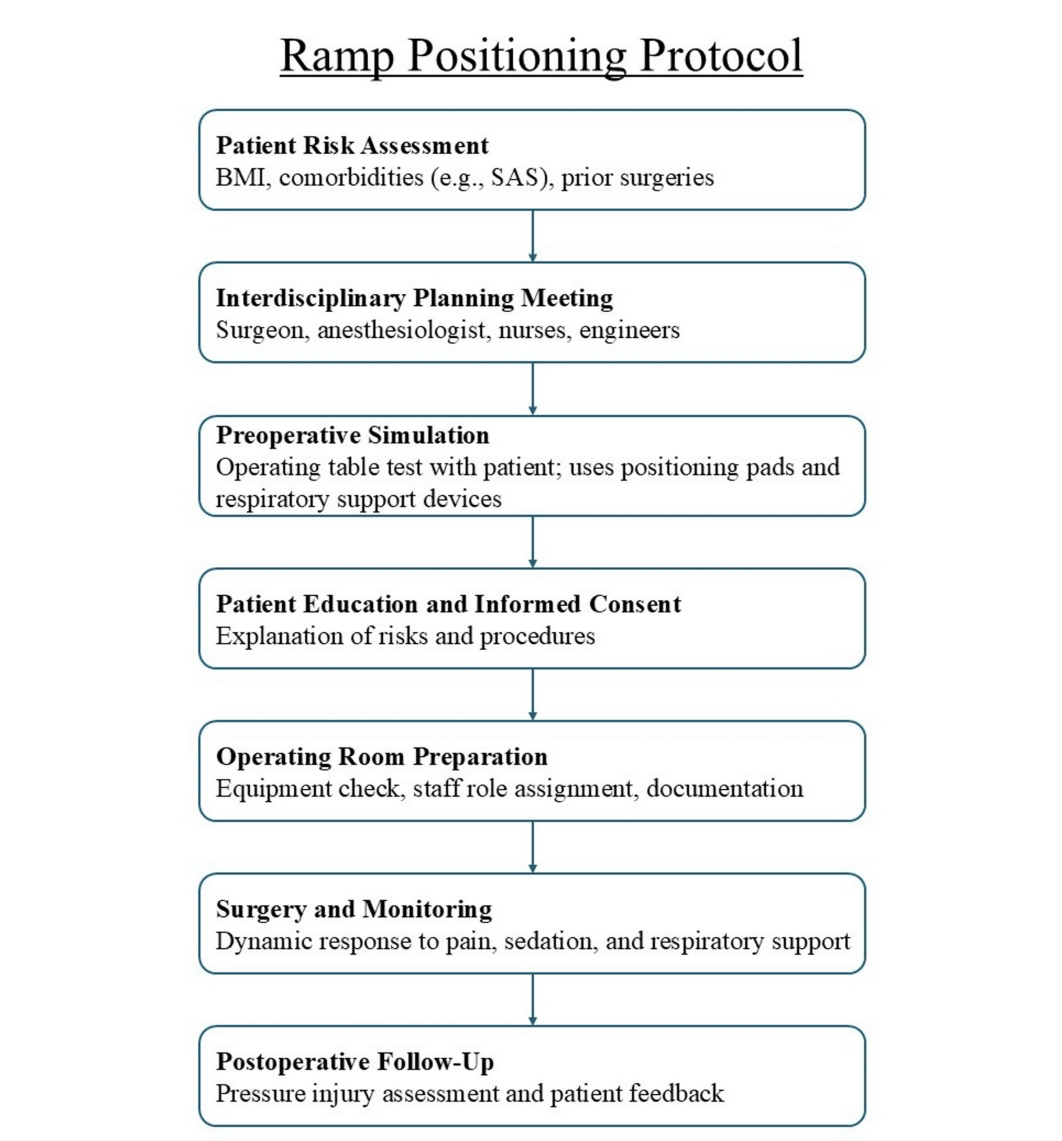
Ramp positioning protocol for morbidly obese parturients undergoing cesarean section A step-by-step flowchart illustrating the clinical workflow for implementing ramp positioning in morbidly obese parturients scheduled for cesarean section. The protocol includes patient risk assessment, interdisciplinary planning, preoperative simulation, informed consent, operating room preparation, intraoperative management, and postoperative evaluation. This structured approach enhances safety, respiratory stability, and surgical access while facilitating communication and role clarity among perioperative team members. SAS: sleep apnea syndrome

To facilitate replication and broader application of this approach, we propose a step-by-step ramp-positioning algorithm (Figure 4), developed based on this case experience. The protocol includes patient assessment, simulation-based validation, equipment preparation, and team coordination steps to ensure safety and operability.

Implementation of this strategy requires certain resources, including simulation access, adjustable operating tables, CPAP/BiPAP machines, and positioning devices such as PureFix®, SliceFix®, and arm/leg supports. Although most are standard in high-resource hospitals, device cost, and personnel availability must be considered when applying this protocol in other settings.

Informed consent was obtained from the patient not only for the surgical procedure and anesthetic plan but also specifically regarding the off-label use of the ramp position, including its potential risks and simulation-based validation. Her participation in the preoperative simulation and agreement to image use were also documented.

This report describes a single case, which inherently limits the generalizability of the findings. Although the patient expressed satisfaction with the simulation-based preparation and individualized care, no validated psychological assessment tools were used to quantify anxiety reduction or emotional burden. Additionally, while the ramp position enabled a safe cesarean delivery, the surgical duration was notably extended compared to average cesarean sections, highlighting procedural complexity in morbidly obese patients. The scalability of simulation-based planning may also be constrained by institutional resources and staffing capacity. These limitations suggest the need for further research involving multiple cases or controlled studies to evaluate the broader applicability and effectiveness of this approach.

## Conclusions

This case report demonstrates that cesarean section in the ramp position can be safely performed in morbidly obese patients when accompanied by thorough preoperative planning and multidisciplinary collaboration. Based on this experience, we recommend that preoperative simulation should be considered for all cesarean deliveries in patients with a BMI of >50, as it enables proactive risk assessment and team coordination.

The approach outlined in this report may also be transferable to other high-risk surgeries involving morbidly obese patients, such as bariatric or orthopedic procedures requiring non-standard positioning. Key resource requirements include access to simulation facilities, availability of adjustable positioning devices, and adequate staffing to ensure safe patient handling. A technical appendix has been added, detailing surgical team composition (including the number and roles of assistants), incision type, and closure methodology, to facilitate replication in similar clinical environments. The successful outcome in this case highlights the critical value of individualized perioperative nursing interventions and interprofessional communication in managing high-risk obstetric patients. Simulation-based preparation serves as a practical model for enhancing surgical safety and patient-centered care in complex cases.
